# Assessment of the Effect of the COVID-19 Pandemic on the Lifestyle of the Population in Saudi Arabia: A Cross-Sectional Online Survey Study

**DOI:** 10.7759/cureus.19796

**Published:** 2021-11-21

**Authors:** Intessar Sultan, Roba A Alobaidi, Khulood K Sewaid, Maryam U Bader, Norah T Almuwallad, Rehab A Mohammed

**Affiliations:** 1 Internal Medicine, Ibn Sina National College for Medical Studies, Jeddah, SAU; 2 Medicine, Ibn Sina National College for Medical Studies, Jeddah, SAU; 3 Medicine and Surgery, Ibn Sina National College for Medical Studies, Jeddah, SAU; 4 Family Medicine, Ibn Sina National College for Medical Studies, Jeddah, SAU; 5 Medical Education and Simulation, Ibn Sina National College for Medical Studies, Jeddah, SAU; 6 Internal Medicine, Faculty of Girls, Al-Azhar University, Cairo, EGY

**Keywords:** s: saudi arabia, fast food, sleeping pattern, smoking habits, dietary habits, physical activity, social practice, life style, corona pandemic, covi-19

## Abstract

Background

The coronavirus (COVID-19) pandemic has created an unprecedented problem in people's lives around the world. Lockdown measures altered the routine lifestyle aspects of people including diet, exercise, sleep, stress, smoking, job status, recreation, and application of modern technologies. Understanding the lifestyle profile of individuals could help in designing effective interventions to minimize the risk factors of COVID-19-related health problems.

Objectives

The aim of this research is to investigate the lifestyle changes among adults living in Saudi Arabia (SA) during the COVID-19 pandemic.

Methods

A cross-sectional survey study was done to investigate the lifestyle changes during the COVID-19 pandemic in Saudi Arabia from August to September 2020. A pre-designed questionnaire was used for data collection and distributed online through social media. The questionnaire included items about sleep patterns, dietary habits, physical activity, employment status, recreation activities, use of social media, and screen time before and during the pandemic.

Results

A total of 338 adults with a median age of 40 years participated in the study. During the pandemic, employment, smoking decreased significantly (53.3 vs. 55.6%, p<0.001 & 15.7% vs. 18.3%, p=0.049) with significant increases in daily intake of fruits or vegetables (47% to 60.2%, p<0.001), caffeinated beverages (seven or more times caffeine, 3% vs. 0.9%, p<0.001), and water (more than eight water cups daily, 18.4 vs. 11.9, p<0.001). Overweight group increased significantly from 28.5% to 32% (p=0.009). Sleeping more than nine hours increased significantly from 8.3% to 21.8% (p<0.001) with increasing sleeping aids from 11.6% to 15.7% (p<0.001). Both the screen and the social media times increased significantly for six or more hours daily (14.8% vs. 35.3% and 9.5% vs. 28.2% respectively, p=<0.001 for both). There were significant decreases in socialization (91.4% vs. 37.8%) and fast food (71.98% vs. 47.04%), and increases in physically inactivity (19% vs. 5.3%) and stress (90.8% vs. 85.2%) (p<0.001 for all). Most participants perceived stress during the pandemic (307, 90.8%).

Conclusion

There were remarkable behavioral changes in all aspects of the lifestyle of the participants living in SA during the COVID-19 pandemic with some positive effects on smoking and dietary habits. However, negative changes included unemployment, physical inactivity, sleep disturbances, social isolation, and excessive weight gain. There is a need to study the possible consequences of such changes on the future population health in SA.

## Introduction

The current coronavirus disease 2019 (COVID-19) pandemic is a public health threat that affects all the world. Multiple preventive measures have been applied by most governments in a trial to limit its spread [1]. Home quarantine was applied in many communities [2], and under such exceptional circumstances, lifestyle has been affected resulting in both physical and mental health problems [3].

Lifestyle is defined as "the characteristics of inhabitants of a region in a special time and place". It has a major impact on health. Variables of lifestyle include diet, exercise, sleep, stress, smoking, and drug abuse, study, recreation, and application of modern technologies [4].

Saudi Arabia (SA) announced the first case of COVID-19 on March 2nd, 2020 [5]. The Ministry of Health in Saudi Arabia has put a lot of work to control the coronavirus spread. The lockdown was implemented by the Saudi government by the end of March 2020 for all its citizens for around three months [6]. Residents were not permitted on the streets, except for experts, such as health care personnel and police officers to control the spread of the virus [5-6]. Daily life was affected with all shops, malls, and restaurants being closed between March and June of 2020. Only supermarkets and bakeries were permitted to open, allowing online selling and in stores with banning serving food or beverages on their premises [6].

The global population is witnessing life-altering challenges due to the COVID-19 pandemic [7]. Given the lack of successful COVID-19 therapy, there is a need for non-pharmacological interventions (NPIs) for the reduction of its transmission [8]. NPIs are intended to adjust lifestyle habits related to illnesses in such a way that they can no longer contribute to the disease's spread. These lifestyle or behavioral changes may lead to mental health effects [9-11], especially those related to personal restrictions, mass confinement, and compulsory home isolation. It was found that pandemic-related coping strategies had an adverse impact on mental health as insomnia, and anger, increases stress, depression, and anxiety [12-14].

The emerging unhealthy lifestyle habits with the pandemic such as poor diet, physical absence, smoking, and alcohol consumption contribute to the global burden of disease and lead to poorer results for mental health [15].

A study done in Kuwait found that skipping breakfast has slightly increased during the pandemic with a drastic decrease in the frequency of fast-food consumption during COVID-19. There was a significant increase in the percentage of participants who had their main meal freshly made. Before and after the pandemic, there were no remarkable improvements in beverage intake, except for American coffee and fresh juice. In addition, there was a substantial decline in physical activity and an increase in screen time and sedentary behaviors. There was an increase in daytime sleepiness and a decrease in night-time sleep [16].

In the Kingdom of Saudi Arabia, a study was done in Riyadh to assess the COVID-19 pandemic’s effect on eating habits among Saudi people. The majority (85.6%) of the participants reported eating homemade food daily during COVID-19 as compared to 35.6% before the pandemic. The quantity of food means' score was higher during the COVID-19 period as compared to the before period. The study determined significant changes in dietary habits among Riyadh residents during the COVID-19 pandemic. Although some good habits increased, the quality and the quantity of food were compromised [17].

Studies assessing lifestyle changes among the Saudi population during the COVID-19 pandemic are limited. The current study aimed to assess the effect of the COVID-19 pandemic on the different lifestyle aspects of adults living in Saudi Arabia.

## Materials and methods

This cross-sectional study was done from August to September 2020 using an online survey. Participants were selected using a non-probability convenient sample. The calculated sample size of 270 subjects. The minimal sample size required according to alpha 5%, and beta 20% and 5 degrees of freedom were 270 individuals. The inclusion criteria were adults who have access to social media and reside in SA and were in the kingdom at times of the COVID-19 pandemic. The exclusion criteria were adults with chronic physical or psychiatric illnesses.

A self-administrated online survey was designed using Google document forms in Arabic language and distributed online through social media. The researchers constructed the survey after an intensive literature review. The questionnaire reliability was assessed using a pilot sample of 20 participants to assess its validity before distribution. The questionnaire included items about employment, smoking (cigarettes, shisha, vape), sleep pattern (duration of sleep, time to sleep, use of sleeping aids, satisfaction after getting up in the morning), dietary habits (vegetables, fruits, caffeinated beverages, water, fast foods), self-reported physical activity, anthropometric measures (weight, height), and the use of social media and screen time among the participants before and during the pandemic. Body mass index (BMI) categories were calculated from height and weight before and during the pandemic. In addition, participants were asked to report their subjective feeling of stress before and during the pandemic.

Ethical approval for the study was obtained from the research ethics committee of Ibn Sina National College for Medical Studies (H-07-13082020 & 007SRC02082020) and an electronic consent was filled by the participants before sharing in the study. In the questionnaire, it was clearly stated that participation was totally voluntary and participants could be withdrawn at any time and that participants’ anonymity would be preserved.

Statistical analysis

**Figure 1:**
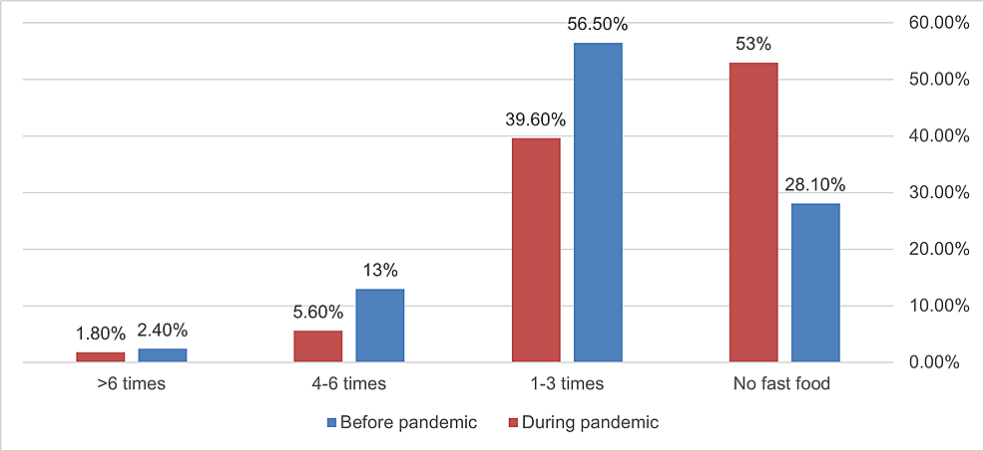
Frequency of weekly fast-food consumption among participants before and during the pandemic (n=338, p <0.001).

**Figure 2:**
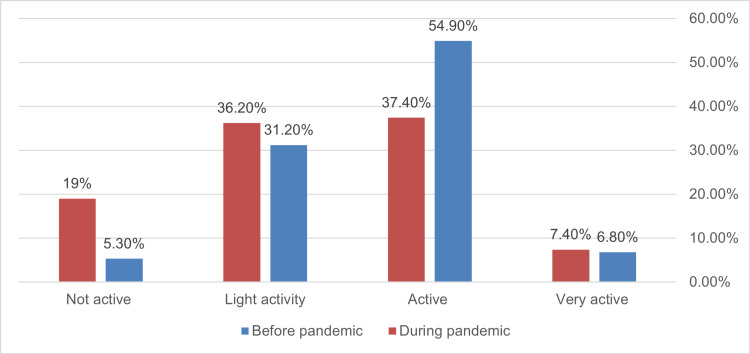
Frequency of weekly fast-food consumption among participants before and during the pandemic (n=338, p <0.001).

**Figure 3:**
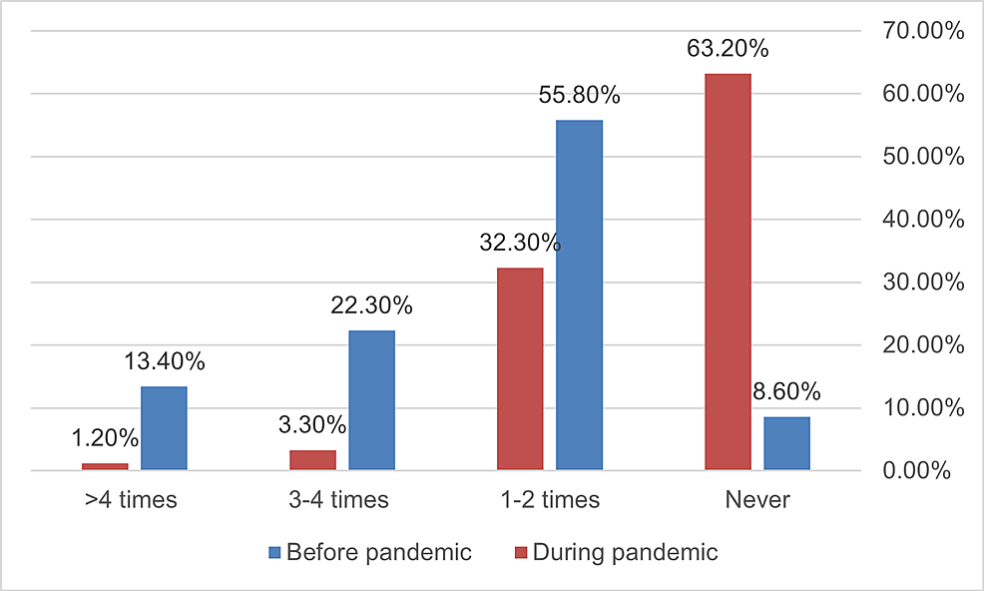
Socialization, or the monthly times of visiting friends and relatives among participants before and during the pandemic (n=338, p <0.001).

**Figure 4:**
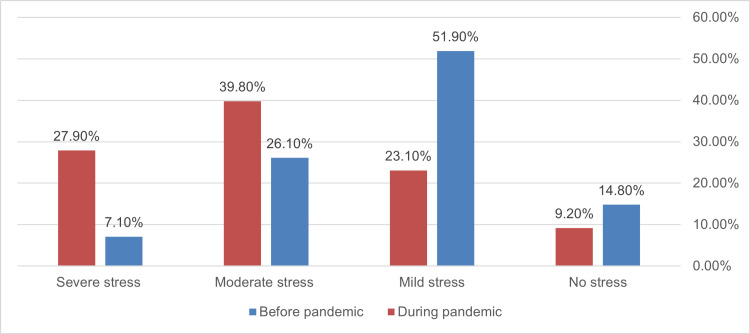
Self-reported stress perception among participants (n=338, p<0.001)

Data were analyzed using SPSS Statistics v. 25 (IBM Corp., Armonk, NY, USA), where qualitative data was expressed as numbers and percentages. The chi-square test was applied to compare variables before and during the pandemic. Microsoft Excel sheets were used to construct Figures 1-4. A p-value of <0.05 was considered statistically significant.

## Results

Three hundred thirty-eight individuals responded to the survey. Participants were 267 females (79%) and 71 males (21%) with a median age of 40 which ranged from 30 to 44 years.

Table 1 shows that the percentage of employment significantly decreased during the pandemic (53.3% vs. 55.6%, p<0.001) as well as the smoking habit (15.7% vs. 18.3%, p=0.049) with 10 individuals reporting that they gave up smoking during the pandemic. Almost all smokers decreased their daily number of cigarettes. Many participants did not go to visit others (63.2%), a significant change to their usual routine (8.6%, p<0.001).

**Table 1:** Comparison of the employment and smoking status before and during the pandemic (n=338)

Variable	Before the pandemic N (%)	During the pandemic N (%)	P
Working status			< 0.001
Employed	188 (55.6)	180 (53.3)	
Unemployed	150 (44.4)	158 (46.7)	
Smoking status			0.049
No	276 (81.7)	285 (84.3)	
Yes	62 (18.3)	52 (15.7)	
Smoking frequency/day			< 0.001
Once	18 (5.3)	14 (4.1)	
2-3 times	17 (5)	17 (5)	
4-5 times	6 (1.8)	2 (0.1)	
> 5 times	21 (6.2)	19 (5.6)	

Table 2 shows that the daily fruit or vegetable intake increased significantly from 47% to 60.2% during the pandemic (p<0.001). Similarly, the daily consumption of caffeinated beverages and cups of water increased significantly especially among those who consume caffeinated beverages seven or more times daily (3% vs. 0.9%, p<0.001) and more than eight cups of water daily (18.4% vs. 11.9%, p<0.001). Self-reported anthropoidal measurements revealed that there was a tendency of weight gain across all Body Mass Index (BMI) categories especially among the overweight group which increased from 28.5% to 32% (p=0.009).

**Table 2:** Comparison of the dietary habits and weight before and during the pandemic (n=338)

Variable	Status before the pandemic N (%)	Status during the pandemic N (%)	P
Daily fruits or vegetable intake			< 0.001
No	159 (52.8)	134 (39.8)	
Yes	178 (47.2)	203 (60.2)	
Daily consumption of caffeinated beverages			0.008
None	51 (15.1)	48 (14.2)	
Once	116 (34.4)	100 (29.7)	
2-3 times	136 (40.4)	142 (42.1)	
4-6 times	31 (9.2)	37 (11)	
≥7 times	3 (0.9)	10 (3)	
Daily number of water cups			< 0.001
<3	96 (28.5)	59 (17.5)	
3-5	118 (35)	108 (32)	
6-8	83 (24.6)	108 (32)	
>8	40 (11.9)	62 (18.4)	
Body Mass Index (BMI) categories			0.009
Underweight	28 (8.3)	22 (6.5)	
Normal weight	115 (34.1)	105 (31.2)	
Overweight	96 (28.5)	108 (32)	
Obese	98 (29.1)	102 (30.3)	

The percentage of participants who used to sleep more than nine hours daily increased significantly during the pandemic from 8.3% to 21.8% (p<0.001). Use of sleeping aids increased from 11.6% to 15.7% (p<0.001), with 7.7% of participants having difficulty falling asleep for more than two hours. Moreover 41.8% did not feel relaxed after getting up from sleep during the pandemic compared to 14.2% before (p<0.001) (Table 3).

**Table 3:** Comparison of the sleeping habits before and during the pandemic (n=338)

Variable	Status before the pandemic N (%)	Status during the pandemic N (%)	P
Daily sleeping hours			< 0.001
<7	192 (57)	106 (31.5)	
7-9	117 (34.7)	161 (47.8)	
>9	28 (8.3)	70 (21.8)	
Sleeping aids			< 0.001
No	298 (88.4)	284 (84.3)	
Yes	39 (11.6)	53 (15.7)	
Time to fall asleep			< 0.001
>2 hours	10 (3)	26 (7.7)	
1 to 2 hours	36 (10.7)	111 (32.9)	
30 minutes to 1 hour	138 (40.9)	116 (34.4)	
<30 minutes	153 (45.4)	84 (24.9)	
Feeling relaxed after waking up			< 0.001
No	48 (14.2)	141 (41.8)	
Yes	289 (85.8)	196 (58.2)	

During the pandemic, both the screen and the social media times increased significantly for six or more hours daily (from 14.8% to 35.3% for screen times and from 9.5% to 28.2% for social media, p=<0.001 for both) (Table 4).

**Table 4:** Comparison of the screen and social media times before and during the pandemic (n=338)

Variable	Status before the pandemic N (%)	Status during the pandemic N (%)	P
Screen time: hours			< 0.001
<1	45 (13.4)	24 (7.1)	
1-3	164 (48.7)	99 (29.4)	
4-5	78 (23.1)	95 (28.2)	
≥6	50 (14.8)	119 (35.3)	
Social media time: hours			< 0.001
<1	58 (17.2)	31 (9.2)	
1-3	158 (46.9)	109 (32.3)	
4-5	89 (26.4)	102 (30.3)	
≥6	32 (9.5)	95 (28.2)	

The total frequency of fast-food consumption dropped significantly during the pandemic from 71.98% to 47.04% (p<0.001) and across all frequencies of consumption (Figure 1). Physical inactivity increased significantly from 5.3% to 19% during the epidemic (p<0.001) (Figure 2). Before the pandemic, more than half of the participants considered themselves active compared to 37.4% during it (p<0.001) (Figure 2). Socialization among participants dropped significantly during the pandemic from 91.4% to only 37.8% (p<0.001) with 63.2% expressing no socialization at all compared to 8.6% before the pandemic (Figure 3).

Most participants perceived stress during (307, 90.8%) compared to before (288, 85.2%) the pandemic. The severity of stress perception increased also significantly especially for severe stress which increased from 7.1% to 27.9% as shown in Figure 4.

## Discussion

We found significant lifestyle changes during the COVID-19 pandemic among our participants, together with increased stress perception. The main positive changes were reported in smoking habits, increased intake of fruits, vegetables, water, and decreased intake of fast food. However, the negative changes exceed the positive ones. The most striking negative changes were in unemployment, physical inactivity, social withdrawal, sleep disturbances, long screen and social media times, and weight gain.

These findings could be easily explained by social distancing and home isolation during the pandemic. Nevertheless, they represent a massive impact on human health, with possible social and economic consequences [18], increases in poverty, and food and nutrition insecurity including young children, adolescents, pregnant and lactating women [19]. The first impact seen in our study was the rise of unemployment from 44% to 46.7%.

Smoking remains a major cause of health hazards. Moreover, smoking is directly linked to poor outcomes of COVID-19 as reported from a large UK Biobank cohort study [20]. This may be the cause of improvement in smoking habits among participants as they might get across to the knowledge from social media. Unhealthy diets; including fast foods, and the associated obesity may act as risk factors for COVID-19 viral infection. During the pandemic, the response of patients with obesity to treatment and even to vaccination is dramatically reduced [21]. 

Moreover, fast foods intake can lead to chronic activation of the intrinsic immune system and an inhibition of the adaptive immune system [22] and it might be linked to defective host protection against viruses.

In this study, significantly increased physical inactivity from 19% was seen but not seen across all levels of activities. It was only reported among those who considered themselves active before the pandemic. Similarly, in another study, the decreased level of activity was not universal, as the group who showed low activity before the pandemic showed some increased activity [23].

During the pandemic quarantine, some cannot work from home and a number even lost their jobs transiently, while others managed to work for a long period from home. The aforementioned groups stay in the home for long periods with increased stress, sleep disturbances, consuming high caloric juices and fruits for obtaining vitamin C, and eating without plans. All these significant behavioral changes might be linked to their increased physical inactivity and result in weight gain. Self-reported measurements of weight and height showed increased categories of overweight and obesity among participants during the pandemic.

Among our participants, sleep was markedly affected by longer hours, more use of sleep aids, longer times to fall asleep, and less satisfaction after getting up from sleep [24]. In concordance with our findings, another study reported that more than half of their participants felt unrested after waking up during the pandemic [25]. Other studies reported a disturbing sleep circadian rhythm during the quarantine probably due to decreased sunlight exposure and increased stress perception [26-27]. The marked disturbance of our participants' sleep-wake-up cycles and sleep quality may be explained by the increased stress perception and the use of sleep aids. However, in contrast to our findings, sleep time was found to decrease during the pandemic and students felt better after sleep as they did not get up early in the early morning to go to school [28].

Because of quarantine, we reported significant social withdrawal and higher screen time especially for engagement in social media. Unfortunately, one alarming study-related increased screen time to the development of depression symptoms [29] which might cause a vicious circle of increasing social withdrawal. Furthermore, another study found that screen time is linked to physical inactivity in association with different degrees of restrictions during the quarantine period [30].

This study has many limitations that limit its generalization. First, the self-reported non-validated online questionnaire, non-randomized sampling method, and the cross-sectional study design. The study information was collected months after quarantine lockdown with possible recall bias. Results should be interpreted cautiously as the majority of the participants were females.

## Conclusions

There were remarkable behavioral changes in all aspects of the lifestyle of the participants living in Saudi Arabia during the COVID-19 pandemic with some positive effects on smoking and dietary habits. However, negative changes included unemployment, physical inactivity, sleep disturbances, social isolation, and overweight. Findings point to the ideal chance of the pandemic to stress the strong ability of the public to fight tobacco and unhealthy food habits when needed. This should be emphasized by the authorities in SA to stress on the public to continue fighting smoking and unhealthy dietary habits even after the pandemic. However, the unwanted behavioral changes adapted during the same pandemic warrant further studies for the possible consequences of such changes on the future population health in Saudi Arabia.
